# Fertility-sparing surgery: a hopeful strategy for young women with cancer

**DOI:** 10.25122/jml-2023-0203

**Published:** 2023-07

**Authors:** Valentin Nicolae Varlas, Roxana Georgiana Borș, Rebeca Crețoiu, Irina Bălescu, Nicolae Bacalbașa, Monica Cîrstoiu

**Affiliations:** 1 Department of Obstetrics and Gynaecology, Filantropia Clinical Hospital, Bucharest, Romania; 2 Department of Obstetrics Gynecology, Faculty of Dentistry, Carol Davila University of Medicine and Pharmacy, Bucharest, Romania; 3 Department of Pituitary and Neuroendocrine Disorders, C.I. Parhon National Institute of Endocrinology, Bucharest, Romania; 4 Department of Surgery, Ponderas Academic Hospital, Bucharest, Romania; 5 Department of Visceral Surgery, Fundeni Clinical Institute, Bucharest, Romania; 6 Department of Obstetrics and Gynecology, University Emergency Hospital Bucharest, Romania

**Keywords:** fertility preservation, oncofertility, fertility-sparing surgery, young cancer patient, computer-assisted surgery, ovarian tissue transplantation, ovarian transposition, robotic surgery

## Abstract

Fertility preservation in cancer patients is currently based on either assisted reproductive technology or fertility-sparing surgery. Loss of fertility may be caused by excisional surgery associated with an adnexal or uterine pathology or secondary to gonadal insufficiency caused by chemotherapy or radiation. The counseling of these patients is very important, being carried out jointly by the oncologist, gynecologist, and reproductive medicine specialist. Reproductive surgery usually requires avoiding laparotomy to significantly reduce the formation of adhesions and trauma or tissue damage. This is done using standard laparoscopic surgery or robotic surgery (computer-assisted laparoscopy), a method increasingly used and accessible to all specialists who want to maintain the fertility of their patients with various oncological diseases.

## INTRODUCTION

Adolescents and young adults (AYA) with cancer are often unaware that their life-preserving treatments can also threaten their future fertility potential [1–3]. Oncofertility (OF) is a growing area due to the increasing number of cancer survivors, the development of new oncologic therapies, the extension of treatments' duration, and the development and refinement of reproductive treatments [4]. The decision to protect fertility from the damaging effects of treatments, like radiation and chemotherapy, is complicated by the patient’s age, marital status, whether they can delay treatment, and, sometimes, the uncertainty of survival [5].

In women, chemotherapy is associated with a decrease in anti-Müllerian hormone (AMH) levels and an increase in follicle-stimulating hormone (FSH) levels, indicating a decline in ovarian reserve and potential impairment of fertility [6, 7]. Decreased estrogen levels leading to symptoms of menopause (such as hot flashes, vaginal dryness, or mood swings) are also frequent [8]. Biological markers serve not only as valuable tools for assessing infertility but also for investigating the recovery of ovarian function. Kim *et al*. concluded that post-chemotherapy AMH levels could be a relatively accurate predictor of ovarian function recovery, as indicated by the resumption of menstruation, in breast cancer patients with chemotherapy-induced amenorrhea [9].

Current studies revealed the risk-stratified priority in cancer patients to optimize care among various malignancies; thus, Smith *et al*. [10] on breast cancer, Wallis *et al*. [11] on urologic cancers, Sica *et al*. [12] and Passamonti *et al*. [13] on onco-hematology, Martinelli *et al*. [14] and Colombo *et al*. [15] on gynecologic cancers.

For patients with oncological diseases, the surgical options that preserve fertility are mainly addressed to four major types of gynecological cancers (cervical, endometrial, ovarian, and breast). In recent years, some recommendations and innovative experimental research have been implemented on fertility conservation techniques in these patients.

For early stages of cervical cancer, cold knife tanning, loop electrosurgical excision procedure (LEEP), simple or radical trachelectomy with a permanent strap and sentinel node mapping, or extra fascial hysterectomy with ovarian preservation is recommended. In advanced diseases where chemotherapy is necessary, preservation of oocytes or ovarian tissue could be performed. Gonadotropin-releasing hormone (GnRH) agonists have also been used for their potential to temporarily suppress ovarian function during cancer treatment, thereby reducing the risk of ovarian damage and preserving fertility. By suppressing ovarian function, GnRH agonists may protect the ovaries from the toxic effects of chemotherapy or radiation therapy [16]. If radiotherapy is needed to preserve ovarian function, ovarian transposition is used before starting [17].

Treatment for the early stages of endometrial cancer requires hysteroscopic tumor resections and progestin therapy with mandatory endometrial sampling every 3-6 months. If cancer regression occurs, therapy will be stopped to allow conception; otherwise, an ovarian preservation hysterectomy is recommended. In the latter case, a gestational carrier can use assisted reproduction technology with the patient's oocytes [18].

In the case of ovarian cancer, conservative surgery includes unilateral/bilateral salpingo-oophorectomy with lymphadenectomy and uterine conservation, depending on the extemporaneous anatomopathological result. If the situation recommends the use of adjuvant chemotherapy, the potential gonadotoxicity could cause a degree of ovarian failure of 3-10%. Due to the increased risk, cryopreservation of oocytes can be considered only in patients with unilateral damage, and in the early stages, an option may be the artificial ovary [19]. This oncological condition requires a lot of additional research.

The progress of fertility-preserving surgical techniques in gynecologic cancers has been associated with progression-free survival rates and overall survival compared with more radical surgeries and in full accordance with patients' reproductive desires without compromising their safety.

For female patients, embryo/oocyte cryopreservation before starting anticancer therapies is the first option to be discussed, but if the chemotherapy is urgent, surgical ovarian tissue cryopreservation may be indicated. A surgical ovarian transposition procedure is recommended before pelvic radiotherapy; temporary ovarian suppression with luteinizing hormone-releasing hormone agonists during the chemotherapy is a simple at-home pharmacological intervention [20]. The next step in oncofertility options includes neoadjuvant cytoprotective pharmacotherapy, fractionation of chemotherapy and radiotherapy, artificial ovary, testicular tissue freezing, and stem cell procedures [21].

The last few years have represented a new way of thinking about fertility surgery in patients with certain types of cancer, both about introducing innovative surgical techniques (radical trachelectomy, ovarian transposition) and some major discoveries such as tissue transplantation, thawed cryopreserved ovary, artificial ovary, and uterine transplant [22]. This research race on new fertility conservation strategies has provided several viable options for young cancer patients with social integration and continuous growth in quality of life. In conclusion, this is a triumph of life over the disastrous consequences of the onset and evolution of cancer.

## MATERIAL AND METHODS

### Data Search

In the present study, we performed a narrative review with a search on the following topics: (1) “fertility preservation,” (2) “oncofertility,” (3) “fertility-sparing surgery,” (4) “cancer patient” - published in peer-reviewed journals and written in English within a ten-year timeframe. We applied the search with filters: Clinical Trial (CT), Randomized Controlled Trial (RCT), and Clinical Case Series (CCS). The review methods of the search were previously established and involved PubMed^®^/MEDLINE database scan.

### Data extraction

The following data were extracted: author (s), year, country of publication, the aim of the study, study design, and main results. Two authors (V.N.V. and R.G.B.) extracted and analyzed the data. Over 100 studies were identified and screened for eligibility. According to the topic search, data extracted included demographic variables, number of participants in the study, treatment, side effects profile, and associated comorbidities. Twenty-nine papers were included in the present study, centered on the main topics included in the search. The statistical analysis used Microsoft Excel^®^ 2013 (Microsoft^®^ Corporation, Redmond, WA, USA).

## RESULTS

### Study selection

Despite the plethora of published papers featuring the keywords “fertility preservation’’ and ‘’cancer patient” during the preliminary search, the PubMed^®^ search retrieved only 2943 all-time results, 2117 from the ten-year span between 2012 and April 2021, with 117 meta-analyses and systematic reviews, and 53 randomized controlled trial and clinical trials. The combined search terms “fertility preservation” and “oncofertility” revealed 337 results, with 4 CT/RCT; “fertility preservation” and “fertility-sparing surgery” retrieved 2250 results with 43 CT/RCT.

**Figure 1:**
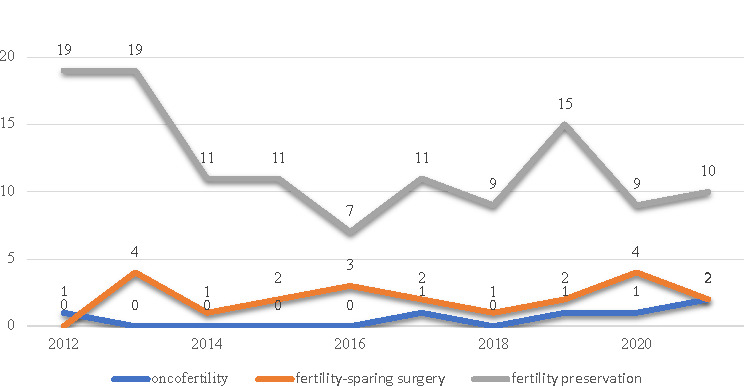
Chart of a search for the keywords “fertility preservation,” “fertility-sparing surgery,” and “oncofertility” on the PubMed® database refined to RCT/CT (ten years topic: 2011-2021)

The evaluation of the last ten years refined to RCT/CT observed the constant interest in young cancer patient topics and a scarcity of data regarding the fertility-sparing surgery and oncofertility topic (Figure 1).

## FERTILITY-SPARING MANAGEMENT IN CANCER PATIENTS

The importance of preserving fertility is given by the overall survival rate of children treated for cancer that exceeds 80%, which explains the growing number of survivors [23]. The therapeutic management of childhood cancer is multimodal and consists of various combinations (surgery, chemotherapy, and radiation therapy). These young patients and their families should be informed about fertility preservation options, such as oocytes or ovarian tissue cryopreservation, in relation to the impact of chemotherapy on gonadal function [24]. Young women diagnosed with cancer, at risk for premature ovarian insufficiency, can choose fertility-sparing surgery (FSS) as an alternative to oocyte or embryo cryopreservation. This technique offers the possibility of having genetic children after the cancer is cured. Ovarian tissue cryopreservation (OTC) is the surgical procedure often used, followed by ovarian tissue transplantation (OTT) at heterotopic or orthotopic sites. Ovarian transposition can be performed in women receiving pelvic radiation [25].

Cryopreservation of ovarian tissue is a technique for preserving fertility addressed to girls and women before initiating gonadotoxic treatments. It is the only possible method for prepubertal girls. The technique was proposed in 1996 and is aimed especially at young patients with various oncological conditions: leukemia, Hodgkin's lymphoma, bone tumors, nephroblastoma, retinoblastoma, and neuroblastoma. It has the advantage that it is immediately available and does not require the administration of hormonal drugs. The preserved ovarian tissue is slow-frozen or vitrified and thus can be used later to restore reproductive and endocrine function in the absence of cancer recurrence [26, 27]. However, there is a significant financial burden associated with fertility preservation, as highlighted by many studies [28–30], with costs ranging from several hundred to several thousand dollars, making it financially challenging for some patients to access these services.

Moreover, the research of Letourneau *et al*. emphasized the time-sensitive nature of fertility preservation, as it requires a delay in cancer treatment initiation [31]. This delay can pose risks, particularly in aggressive malignancies where immediate treatment is crucial. Regarding the success rates, the likelihood of achieving a successful pregnancy with cryopreserved gametes or embryos may vary [32–34] because most patients do not utilize their cryopreserved oocytes [35, 36], which imposes limitations on conducting future studies regarding clinical outcomes.

Ovarian transposition is a surgical procedure that allows fertility preservation and is recommended for women with gynecological oncological conditions (cervical cancer, vaginal cancer) and urological or hematological diseases that require pelvic or craniospinal radiotherapy [26]. Although potential functional decline must also be considered, most studies [37–39] show that ovarian function is preserved and that most transposed ovaries remain metastasis-free. A recent meta-analysis investigated the data of 1160 women with cervical cancer who were subjected to ovarian transposition and found that 93% of women who underwent surgery with or without brachytherapy had their ovarian function preserved, and metastases were presented in the transposed ovaries in only 1% of cases [40]. However, there is scarce literature on the long-term outcomes of ovarian transposition, which poses challenges in accurately assessing its effectiveness and emphasizes the need for more research to evaluate the long-term impact of ovarian transposition on fertility and hormonal function in cancer survivors. Laparoscopic ovarian transposition has a success rate of 88.6% for maintaining ovarian function [41]. Other FSS include uterine fixation, which helps protect against pelvic radiation effects and preserve fertility [42].

Given the secondary gonadotoxicity of aggressive neoadjuvant chemotherapy regimens, the time factor defines these patients, who tend to lose their fertile potential quickly. Thus, cancer patients must start their systemic anticancer treatment immediately, in parallel with evaluating the opportunity to initiate oncofertility-related procedures. The fertile prognostic of young cancer patients was negatively influenced by less surgical training in oncologic surgery and few oncology clinical trials [43].

### Fertility-sparing surgery in cervical cancer

Patients with cervical cancer with tumors <2 cm (IA2-IB2 FIGO) undergoing the radical vaginal trachelectomy - Dargent procedure have a survival rate identical to those undergoing radical hysterectomy. Patients with tumors >2 cm cannot be prescribed the Dargent procedure due to the increased risk of central pelvic recurrence (approximately 17%). The need for parametrectomy is debatable in patients at low risk of parametric invasion due to urinary and digestive complications and the subsequent risk of miscarriage or premature birth [44].

According to the NCCN, for stage IA1 without lymphovascular space invasion (LVSI), simple cone resection or hysterectomy is recommended. In contrast, for stages IA1 with LVSI, IA2 - IB1, simple/radical trachelectomy in association with sentinel node biopsy or pelvic lymph dissection is indicated [45]. The SEER database in a select group of patients with stage IB1 cervical cancer did not show differences in survival between patients treated with less radical surgery and those treated with radical surgery [46].

Plante *et al*. reported a >97% overall survival rate or absence of progression at 5 years in patients with tumors <2 cm who opted for fertility preservation through simple trachelectomy or cone resection with laparoscopic identification of the sentinel node ± pelvic lymph dissection [47]. Bentivegna *et al*. indicated radical trachelectomy in the presence of IB1 stage LVSI [48]. In contrast, in the absence of LSVI, the ConCerv trial showed that in the early stages (tumor <2 cm, histology, depth of invasion <10 mm, and conical biopsy with negative edges), recurrent disease within 2 years of surgery (cone resection or simple hysterectomy) was 3% [49].

The correct selection of patients (tumor size, non-aggressive histological types, lack of lymph invasion, negative lymph nodes), the experience of the surgical team, the correct choice of surgical procedure, the technical possibilities of the center of excellence in gynecological oncological surgery, are elements of favorable prognosis on the preservation of fertility.

Patients who have received FSS for cervical cancer may later experience several risk factors, such as decreased fertility rate, increased risk of miscarriage, cervical failure, risk of chorioamnionitis, premature rupture of membranes, and the onset of preterm labor [50]. If cervical cancer is diagnosed in the first trimester of pregnancy, patients should be properly counseled about the risk of continuing the pregnancy and the appropriate cancer treatment so as not to compromise either the maternal or fetal prognosis [51]. The intraoperative cerclage placed transabdominal either during FSS or subsequent pregnancy is a decision that must be made according to the patient's obstetrical history because performing a simple trachelectomy does not routinely require this procedure [52, 53]. Performing Pap tests and/or colposcopy in patients with FSS in the first trimester in pregnant women is extremely important in diagnosing and monitoring these patients.

In patients who have undergone cone resection, a vaginal birth may be attempted in the absence of residual disease or obstetrical contraindications, as opposed to trachelectomy in which a cesarean section must be performed as the small scarred cervix is at risk of rupture. [47, 54]. Thus, vaginal delivery may be complicated by severe bleeding, rupture of the uterus, or possible metastatic spread in unidentified recurrences [51].

### Fertility-sparing surgery in endometrial cancer

Endometrial cancer (EC) is the fourth most common cancer in women, being the most frequent gynecological cancer [55]. Conservative treatment for fertility preservation in patients with endometrial cancer stage IA FIGO (without or less than half invasion of the myometrium) consists of hormone therapy (progestin-only pills, LNG-IUD), especially in patients with estrogen receptor expression and progesterone combined or not with hysteroscopic endometrial focal resection [56, 57].

In a review of the literature, Garzon *et al*. evaluated the safety of fertility in young women with atypical endometrial hyperplasia (AEH) or grade 1 endometrial cancer (EC). The EC's oral treatment with progestin (medroxyprogesterone acetate and megestrol acetate) is accompanied by a recurrence rate of 30.7% and a pregnancy rate of 52.1%. Other treatment options include LNG-IUD, megestrol acetate plus metformin [58, 59], and hysteroscopic resection followed by progestins [56]. Thus, the latter combination showed a similar response regarding the birth rate of live newborns compared to progestogen monotherapy but with a reduced relapse rate [57]. Gullo's team is more reserved about metformin use and hysteroscopic evaluation, noting that new research is needed [60]. Novikova *et al*., on a group of 418 patients with AEH and grade 1-2 endometrioid EC with minimal or no myometrial invasion on MRI, shows that hormone therapy with LNG-IUD is better than the MPA-containing regimen [61].

Despite this evidence, the lack of randomized clinical trials on the efficacy and safety of treatment in EC in relation to the risk of worsening oncological disease explains the cautious way to preserve fertility in these patients [62]. For obese patients with EC and AEH, a weight loss of over 10% and the administration of GnRH agonists would have an increased effect compared to progestin therapy [63].

GnRH agonists, such as leuprolide acetate, goserelin, and triptorelin, can be used to induce a temporary menopausal state, suppress endogenous estrogen production, and potentially preserve fertility. By reducing the secretion of luteinizing hormone (LH) and follicle-stimulating hormone (FSH) from the pituitary gland, GnRH agonists effectively suppress the ovarian production of estrogen, which is essential for the growth and progression of endometrial cancer. Lower estrogen levels create an unfavorable hormonal environment for cancer cells, potentially leading to disease regression and preservation of fertility [64].

Metabolic syndrome and insulin resistance are associated with an increased recurrence rate in patients with AEH and EC who have resorted to fertility-sparing procedures [65].

After primary conservative treatment for patients with recurrent EC and EAH who wish to preserve their uterus after complete remission, resumption of treatment that preserves fertility may be an option as it allows the completion of pregnancy and delivery [66].

### Fertility-sparing surgery in ovarian cancer

Ovarian cancer is the most aggressive and with the highest mortality among gynecological cancers [67]. Fertility-sparing treatment is used for patients with limited epithelial ovarian cancer (EOC) or a non-epithelial tumor. This also applies to cases with peritoneal implants at the time of surgery. In the case of unilateral borderline mucinous ovarian tumors, the intervention is initial unilateral salpingo-oophorectomy, and in the case of serous ovarian tumors, cystectomy. Unilateral oophorectomy and contralateral ovarian cystectomy may be performed for bilateral borderline ovarian tumors [19].

After staging, FSS in patients with EOC will address stage IA of grade 1/2 of serous, mucinous, or endometrioid tumors or stage IC grade 1. For EOC in stage IA grade 3 or IC1/IC2 grade 1, an option is given by bilateral salpingo-oophorectomy, preservation of the uterus, and the use of donated oocytes [19]. In patients with stage IB, IC2, and IC3 grade 3 or bilateral ovarian impairment, the safety regarding fertility preservation procedures is uncertain about the oncological prognosis. For stages II/III (regardless of histological form), FSS is contraindicated [48, 68]. Another study revealed that the surgical management in these cases is represented by unilateral salpingo-oophorectomy with pelvic and para-aortic lymphadenectomy, peritoneal washing, and omentectomy [68].

Two retrospective studies of 79 patients and 27 patients with non-epithelial cancers (ovarian germ cell malignancies are frequent in reproductive age), regardless of tumor stage and histological type, showed that fertility preservation surgery with adjuvant chemotherapy was followed by a high fertility rate [69, 70]. The rate of live births in patients who wanted to become pregnant ranged from 73% to 80.9% [70, 71]. Furthermore, Tamauchi *et al*., using a multicenter database, identified 56 babies born to 40 malignant ovarian germ cell tumor (MOGCT) survivors from 110 MOGCT patients [72].

Sexually stromal cord tumors diagnosed in young women benefit from FSS, with a risk of premature ovarian failure, a situation that would require cryopreservation of ovarian tissue [73]. The AGO group's CORSETT study found that in addition to preserving fertility, FSS techniques increase the quality of life and do not alter sexuality in patients with non-epithelial ovarian tumors [74].

### Fertility-sparing surgery in vulvar cancer

Vulvar cancer accounts for 4% of gynecological malignancies, with a peak incidence of in situ vulvar carcinoma in the 40-49 age group. The anatomopathological form in women under 45 is vulvar squamous cell cancer [75, 76]. Therapeutic management of these patients concerning fertility preservation should include less destructive surgery without affecting prognosis, cryopreservation of ovarian tissue before chemotherapy, and oophoropexy before pelvic radiotherapy [77]. Diken *et al*. presented a case of a 33-year-old woman diagnosed with synovial cell sarcoma of the vulva. Following radical hemi-vulvectomy with bilateral lymph dissection and subsequent brachytherapy (20 Gy) and external radiotherapy (50 Gy), her fertility was preserved, leading to a later successful childbirth [78].

### Fertility-sparing surgery in breast cancer

In patients under 40, the incidence of breast cancer is over 7% of all oncological conditions. The main options for fertility preservation in women with breast cancer are oocyte or embryo cryopreservation. Also, patients who want to maintain their fertility before breast radiotherapy have two options: either to use shields for the adnexal area or to transpose the ovaries, although the radiation doses that reach the genital organs are small without affecting ovarian function. Some women choose mastectomy as their first intention, precisely to avoid radiotherapy and its possible effects on fertility [79, 80]. Obstetric results after breast cancer treatment are very good [80]. In the case of breast cancer, it seems that the administration of GnRH analogs has a protective effect on maintaining ovarian function in relation to gonadotoxicity secondary to the treatment.

A recent systematic review by Lambertini *et al*. [81] aimed to assess the efficacy and safety of temporary ovarian suppression using GnRH agonists during chemotherapy in premenopausal women with early breast cancer and found that this therapy appears to be an effective and safe option for premenopausal patients with early breast cancer. The results indicate that their addition to the chemotherapy regimen reduces the risk of premature ovarian insufficiency and may increase the likelihood of achieving a pregnancy after treatment. Importantly, this approach did not show any negative impact on disease-free survival or overall survival outcomes. These findings support the use of GnRH analogs as a potential strategy to preserve ovarian function, enhance future fertility prospects, and improve the overall quality of life for premenopausal women undergoing chemotherapy for early breast cancer.

However, many authors argue that the available evidence is insufficient to determine the impact of GnRH agonists on fertility preservation, and further investigation is needed [82–84]. The study of Chen *et al*. [85] suggested that although GnRH agonists can help protect ovarian function during chemotherapy, leading to improved menstruation, reduced premature ovarian failure, and increased ovulation, more research is needed to fully understand their impact on fertility preservation and further studies should explore different age groups, chemotherapy regimens, and long-term outcomes.

## CONCLUSION

Delaying a pregnancy or lowering the age of onset of cancer has revolutionized medicine by developing various surgical interventions to preserve fertility. The correct intervention choice depends on the cancer's location, type, and stage. Thus, regarding the chances related to fertility, an important role belongs to the counselors, a process that must be initiated by the gynecologist and continued by the surgeon, oncologist, chemotherapist, radiotherapist, and psychologist so that in the end, the integrative role to belong to the specialist of reproductive medicine that will balance the benefits versus risks for each case. This is how the multidisciplinary approach to oncofertility is defined. Fertility-sparing surgery has been, is, and will be an additional challenge for cancer care and fertility. The influence can be found at the level of any link within this multidisciplinary team, mainly in initiating aggressive gonadotoxic therapy.

## References

[1] Lobo RA (2005). Potential Options for Preservation of Fertility in Women. N Engl J Med.

[2] Wallace WHB, Smith AG, Kelsey TW, Edgar AE, Anderson RA (2014). Fertility Preservation for Girls and Young Women with Cancer: Population-Based Validation of Criteria for Ovarian Tissue Cryopreservation. Lancet Oncol.

[3] Jeruss JS, Woodruff TK (2009). Preservation of Fertility in Patients with Cancer. N Engl J Med.

[4] Walsh SK, Ginsburg ES, Lehmann LS, Partridge AH (2017). Oncofertility: Fertile Ground for Conflict Between Patient Autonomy and Medical Values. Oncologist.

[5] Woodruff TK (2015). Oncofertility: A Grand Collaboration between Reproductive Medicine and Oncology. Reprod Camb Engl.

[6] Dewailly D, Andersen CY, Balen A, Broekmans F (2014). The Physiology and Clinical Utility of Anti-Müllerian Hormone in Women. Hum Reprod Update.

[7] Cedars MI (2022). Evaluation of Female Fertility—AMH and Ovarian Reserve Testing. J. Clin. Endocrinol. Metab.

[8] Soewoto W, Agustriani N (2023). Estradiol Levels and Chemotherapy in Breast Cancer Patients: A Prospective Clinical Study. World J Oncol.

[9] Kim H-A, Choi J, Park CS, Seong M-K (2018). Post-Chemotherapy Serum Anti-Müllerian Hormone Level Predicts Ovarian Function Recovery Endocr Connect.

[10] Smith BL, Nguyen A, Korotkin JE, Kelly BN (2020). A System for Risk Stratification and Prioritization of Breast Cancer Surgeries Delayed by the COVID-19 Pandemic: Preparing for Re-Entry. Breast Cancer Res Treat.

[11] Wallis CJD, Novara G, Marandino L, Bex A (2020). Risks from Deferring Treatment for Genitourinary Cancers: A Collaborative Review to Aid Triage and Management During the COVID-19 Pandemic. Eur Urol.

[12] Sica A, Casale D, Rossi G, Casale B (2020). The Impact of the SARS-CoV-2 Infection, with Special Reference to the Hematological Setting. J Med Virol.

[13] Passamonti F, Cattaneo C, Arcaini L, Bruna R (2020). Clinical Characteristics and Risk Factors Associated with COVID-19 Severity in Patients with Haematological Malignancies in Italy: A Retrospective, Multicentre, Cohort Study. Lancet Haematol.

[14] Martinelli F, Garbi A (2020). Change in practice in gynecologic oncology during the COVID-19 pandemic: a social media survey. Int J Gynecol Cancer.

[15] Colombo I, Zaccarelli E, Del Grande M, Tomao F (2020). ESMO Management and Treatment Adapted Recommendations in the COVID-19 Era: Gynaecological Malignancies. ESMO Open.

[16] Poggio F, Lambertini M, Bighin C, Conte B (2019). Potential Mechanisms of Ovarian Protection with Gonadotropin-Releasing Hormone Agonist in Breast Cancer Patients: A Review. Clin Med Insights Reprod Health.

[17] Silvestris E, Paradiso AV, Minoia C, Daniele A (2022). Fertility Preservation Techniques in Cervical Carcinoma. Medicine (Baltimore).

[18] Rodolakis A, Scambia G, Planchamp F, Acien M (2023). ESGO/ESHRE/ESGE Guidelines for the Fertility-Sparing Treatment of Patients with Endometrial Carcinoma. Hum Reprod Open.

[19] Canlorbe G, Chabbert-Buffet N, Uzan C (2021). Fertility-Sparing Surgery for Ovarian Cancer. J Clin Med.

[20] Sirohi B, Rohatgi TB, Lambertini M (2020). Oncofertility and COVID-19-Cancer Does Not Wait. Ecancermedicalscience.

[21] Salama M, Ataman-Millhouse L, Braham M, Berjeb K (2020). Installing Oncofertility Programs for Common Cancers in Limited Resource Settings (Repro-Can-OPEN Study): An Extrapolation during the Global Crisis of Coronavirus (COVID-19) Pandemic. J Assist Reprod Genet.

[22] Christianson MS, Oktay K (2019). Advances in Fertility-Preservation Surgery: Navigating New Frontiers. Fertil Steril.

[23] Key Statistics for Childhood Cancers. https://www.cancer.org/cancer/types/cancer-in-children/key-statistics.html.

[24] Kim H, Kim H, Ku SY (2018). Fertility Preservation in Pediatric and Young Adult Female Cancer Patients. Ann Pediatr Endocrinol Metab.

[25] Female Fertility Preservation Guideline of the European Society of Human Reproduction and Embryology 2020. http://www.Eshre.Eu/Guidelines.

[26] Poirot C, Brugieres L, Yakouben K, Prades-Borio M (2019). Ovarian Tissue Cryopreservation for Fertility Preservation in 418 Girls and Adolescents up to 15 Years of Age Facing Highly Gonadotoxic Treatment. Twenty Years of Experience at a Single Center. Acta Obstet Gynecol Scand.

[27] Gjeterud J, Kristensen SG, Fedder J (2021). Indications for Cryopreservation and Autotransplantation of Ovarian Tissue. Tidsskr Den Nor Laegeforening Tidsskr Prakt Med. Ny Raekke.

[28] Benedict C, Thom B, Kelvin JF (2016). Fertility preservation and cancer: challenges for adolescent and young adult patients. Curr Opin Support Palliat Care.

[29] Chung EH, Lim SL, Myers E, Moss HA, Acharya KS (2021). Oocyte Cryopreservation versus Ovarian Tissue Cryopreservation for Adult Female Oncofertility Patients: A Cost-Effectiveness Study. J Assist Reprod Genet.

[30] Lyttle Schumacher B, Grover N, Mesen T, Steiner A, Mersereau J (2017). Modeling of Live-Birth Rates and Cost-Effectiveness of Oocyte Cryopreservation for Cancer Patients Prior to High-and Low-Risk Gonadotoxic Chemotherapy. Hum Reprod.

[31] Letourneau JM, Melisko ME, Cedars MI, Rosen MP (2011). A Changing Perspective: Improving Access to Fertility Preservation. Nat Rev Clin Oncol.

[32] Porcu E, Cipriani L, Dirodi M, De Iaco P (2022). Successful Pregnancies, Births, and Children Development Following Oocyte Cryostorage in Female Cancer Patients During 25 Years of Fertility Preservation. Cancers.

[33] Specchia C, Baggiani A, Immediata V, Ronchetti C (2019). Oocyte Cryopreservation in Oncological Patients: Eighteen Years Experience of a Tertiary Care Referral Center. Front Endocrinol.

[34] Walker Z, Lanes A, Ginsburg E (2022). Oocyte Cryopreservation Review: Outcomes of Medical Oocyte Cryopreservation and Planned Oocyte Cryopreservation. Reprod Biol Endocrinol.

[35] Blakemore JK, Grifo JA, DeVore SM, Hodes-Wertz B, Berkeley AS (2021). Planned Oocyte Cryopreservation—10–15-Year Follow-up: Return Rates and Cycle Outcomes. Fertil Steril.

[36] Mayeur A, Puy V, Windal V, Hesters L (2021). Live Birth Rate after Use of Cryopreserved Oocytes or Embryos at the Time of Cancer Diagnosis in Female Survivors: A Retrospective Study of Ten Years of Experience. J Assist Reprod Genet.

[37] Gubbala K, Laios A, Gallos I, Pathiraja P (2014). Outcomes of Ovarian Transposition in Gynaecological Cancers; a Systematic Review and Meta-Analysis. J Ovarian Res.

[38] Jung W, Kim YH, Kim KS (2021). Ovarian Function Preservation in Patients With Cervical Cancer Undergoing Hysterectomy and Ovarian Transposition Before Pelvic Radiotherapy. Technol Cancer Res Treat.

[39] Arian SE, Goodman L, Flyckt RL, Falcone T (2017). Ovarian Transposition: A Surgical Option for Fertility Preservation. Fertil Steril.

[40] Laios A, Otify M, Papadopoulou A, Gallos ID, Ind T (2022). Outcomes of Ovarian Transposition in Cervical Cancer; an Updated Meta-Analysis. BMC Womens Health.

[41] Arian SE, Goodman L, Flyckt RL, Falcone T (2017). Ovarian Transposition: A Surgical Option for Fertility Preservation. Fertil Steril.

[42] Christianson MS, Oktay K (2019). Advances in Fertility-Preservation Surgery: Navigating New Frontiers. Fertil Steril.

[43] Dellino M, Minoia C, Paradiso AV, De Palo R, Silvestris E (2020). Fertility Preservation in Cancer Patients During the Coronavirus (COVID-19) Pandemic. Front Oncol.

[44] Mejia-Gomez J, Feigenberg T, Arbel-Alon S, Kogan L, Benshushan A (2012). Radical Trachelectomy: A Fertility-Sparing Option for Early Invasive Cervical Cancer. Isr Med Assoc J.

[45] Abu-Rustum NR, Yashar CM, Bean S, Bradley K, Campos SM, Chon HS, Chu C, Cohn D, Crispens MA, Damast S (2020). NCCN Guidelines Insights: Cervical Cancer, Version 1.2020. J Natl Compr Cancer Netw.

[46] Tseng JH, Aloisi A, Sonoda Y, Gardner GJ (2018). Long-Term Oncologic Outcomes of Uterine-Preserving Surgery in Young Women With Stage Ib1 Cervical Cancer. Int J Gynecol Cancer.

[47] Plante M, Renaud MC, Sebastianelli A, Gregoire J (2020). Simple Vaginal Trachelectomy in Women with Early-Stage Low-Risk Cervical Cancer Who Wish to Preserve Fertility: The New Standard of Care?. Int J Gynecol Cancer.

[48] Bentivegna E, Maulard A, Pautier P, Chargari C (2016). Fertility Results and Pregnancy Outcomes after Conservative Treatment of Cervical Cancer: A Systematic Review of the Literature. Fertil Steril.

[49] Schmeler KM, Pareja R, Lopez Blanco A, Humberto Fregnani J (2021). ConCerv: A Prospective Trial of Conservative Surgery for Low-Risk Early-Stage Cervical Cancer. Int J Gynecol Cancer.

[50] Šimják P, Cibula D, Pařízek A, Sláma J (2020). Management of Pregnancy after Fertility-Sparing Surgery for Cervical Cancer. Acta Obstet Gynecol Scand.

[51] Halaska MJ, Drochytek V, Shmakov RG, Amant F (2021). Fertility Sparing Treatment in Cervical Cancer Management in Pregnancy. Best Pract Res Clin Obstet Gynaecol.

[52] Davenport SM, Jackson AL, Herzog TJ (2016). Cerclage during Trachelectomy for Early-Stage Cervical Cancer. Gynecol Oncol.

[53] Rob L, Skapa P, Robova H (2011). Fertility-Sparing Surgery in Patients with Cervical Cancer. Lancet Oncol.

[54] Brătilă E, Brătilă CP, Coroleuca CB (2016). Radical Vaginal Trachelectomy with Laparoscopic Pelvic Lymphadenectomy for Fertility Preservation in Young Women with Early-Stage Cervical Cancer. Indian J Surg.

[55] American Cancer Society (2022). Cancer Facts and Figures 2022. American Cancer Society.

[56] Garzon S, Uccella S, Zorzato PC, Bosco M (2021). Fertility-Sparing Management for Endometrial Cancer: Review of the Literature. Minerva Med.

[57] Giampaolino P, Di Spiezio Sardo A, Mollo A, Raffone A (2019). Hysteroscopic Endometrial Focal Resection Followed by Levonorgestrel Intrauterine Device Insertion as a Fertility-Sparing Treatment of Atypical Endometrial Hyperplasia and Early Endometrial Cancer: A Retrospective Study. J Minim Invasive Gynecol.

[58] Mitsuhashi A, Sato Y, Kiyokawa T, Koshizaka M (2016). Phase II Study of Medroxyprogesterone Acetate plus Metformin as a Fertility-Sparing Treatment for Atypical Endometrial Hyperplasia and Endometrial Cancer. Ann Oncol Off J Eur Soc Med Oncol.

[59] Mitsuhashi A, Shozu M (2020). New Therapeutic Approaches for the Fertility-Sparing Treatment of Endometrial Cancer. J Obstet Gynaecol Res.

[60] Gullo G, Etrusco A, Cucinella G, Perino A, Chiantera V, Laganà AS, Tomaiuolo R, Vitagliano A, Giampaolino P, Noventa M (2021). Fertility-Sparing Approach in Women Affected by Stage I and Low-Grade Endometrial Carcinoma: An Updated Overview. Int J Mol Sci.

[61] Novikova OV, Nosov VB, Panov VA, Novikova EG (2021). Live Births and Maintenance with Levonorgestrel IUD Improve Disease-Free Survival after Fertility-Sparing Treatment of Atypical Hyperplasia and Early Endometrial Cancer. Gynecol Oncol.

[62] Contreras NA, Sabadell J, Verdaguer P, Julià C, Fernández-Montolí ME (2022). Fertility-Sparing Approaches in Atypical Endometrial Hyperplasia and Endometrial Cancer Patients: Current Evidence and Future Directions. Int J Mol Sci.

[63] Chen J, Cao D, Yang J, Yu M (2022). Fertility-Sparing Treatment for Endometrial Cancer or Atypical Endometrial Hyperplasia Patients With Obesity. Front Oncol.

[64] Emons G, Gründker C (2021). The Role of Gonadotropin-Releasing Hormone (GnRH) in Endometrial Cancer. Cells.

[65] Li X, Fan Y, Wang J, Zhou R (2021). Insulin Resistance and Metabolic Syndrome Increase the Risk of Relapse For Fertility Preserving Treatment in Atypical Endometrial Hyperplasia and Early Endometrial Cancer Patients. Front Oncol.

[66] Chen J, Cao D, Yang J, Yu M (2021). Management of Recurrent Endometrial Cancer or Atypical Endometrial Hyperplasia Patients After Primary Fertility-Sparing Therapy. Front Oncol.

[67] Cancer Facts & Figures 2023| American Cancer Society. https://www.cancer.org/research/cancer-facts-statistics/all-cancer-facts-figures/2023-cancer-facts-figures.html.

[68] Bentivegna E, Morice P, Uzan C, Gouy S (2016). Fertility-Sparing Surgery in Epithelial Ovarian Cancer. Future Oncol Lond Engl.

[69] Mikuš M, Benco N, Matak L, Planinić P, Ćorić M, Lovrić H, Radošević V, Puževski T, Bajt M, Vujić G (2020). Fertility-Sparing Surgery for Patients with Malignant Ovarian Germ Cell Tumors: 10 Years of Clinical Experience from a Tertiary Referral Center. Arch Gynecol Obstet.

[70] Zamani N, Rezaei Poor M, Ghasemian Dizajmehr S, Alizadeh S, Modares Gilani M (2021). Fertility Sparing Surgery in Malignant Ovarian Germ Cell Tumor (MOGCT): 15 Years Experiences. BMC Womens Health.

[71] Ertas IE, Taskin S, Goklu R, Bilgin M (2014). Long-Term Oncological and Reproductive Outcomes of Fertility-Sparing Cytoreductive Surgery in Females Aged 25 Years and Younger with Malignant Ovarian Germ Cell Tumors. J Obstet Gynaecol Res.

[72] Tamauchi S, Kajiyama H, Yoshihara M, Ikeda Y (2018). Reproductive Outcomes of 105 Malignant Ovarian Germ Cell Tumor Survivors: A Multicenter Study. Am J Obstet Gynecol.

[73] Di Tucci C, Casorelli A, Morrocchi E, Palaia I (2017). Fertility Management for Malignant Ovarian Germ Cell Tumors Patients. Crit Rev Oncol Hematol.

[74] Hasenburg A, Plett H, Krämer B, Braicu E (2021). The Effect of Fertility-Sparing Surgery on Sexuality and Global Quality of Life in Women with Malignant Ovarian Germ Cell and Sex Cord Stromal Tumors: An Analysis of the CORSETT Database of the AGO Study Group. Arch Gynecol Obstet.

[75] Judson PL, Habermann EB, Baxter NN, Durham SB, Virnig BA (2006). Trends in the Incidence of Invasive and in Situ Vulvar Carcinoma. Obstet Gynecol.

[76] Lanneau GS, Argenta PA, Lanneau MS, Riffenburgh RH (2009). Vulvar Cancer in Young Women: Demographic Features and Outcome Evaluation. Am J Obstet Gynecol.

[77] Narducci F, Sabban F, Vanlerenberghe E, Lesoin A (2006). [What is new in the surgical treatment of pelvic gynecologic cancers?]. Bull Cancer (Paris).

[78] Dicken CL, Lieman HJ, Dayal AK, Mutyala S, Einstein MH (2010). A Multidisciplinary Approach to Fertility-Sparing Therapy for a Rare Vulvar Tumor. Fertil Steril.

[79] Beyer S, Sandu A, White J (2020). Impact and Timing of Breast Cancer Radiation Therapy and Fertility Preservation. Curr Breast Cancer Rep.

[80] Basta P, Streb J, Szczygieł K (2015). Fertility-Sparing Treatment in Female Genital Cancer and Breast Cancer. Ginekol Pol.

[81] Lambertini M, Moore HCF, Leonard RCF, Loibl S (2018). Gonadotropin-Releasing Hormone Agonists During Chemotherapy for Preservation of Ovarian Function and Fertility in Premenopausal Patients With Early Breast Cancer: A Systematic Review and Meta-Analysis of Individual Patient–Level Data. J Clin Oncol.

[82] Munhoz RR, Pereira AAL, Sasse AD, Hoff PM (2016). Gonadotropin-Releasing Hormone Agonists for Ovarian Function Preservation in Premenopausal Women Undergoing Chemotherapy for Early-Stage Breast Cancer: A Systematic Review and Meta-Analysis. JAMA Oncol.

[83] Choi MC, Chung YS, Lee JW, Kwon BS (2020). Feasibility and Efficacy of Gonadotropin-Releasing Hormone Agonists for the Prevention of Chemotherapy-Induced Ovarian Insufficiency in Patients with Malignant Ovarian Germ Cell Tumours (KGOG 3048R). Eur J Cancer.

[84] Turner NH, Partridge A, Sanna G, Di Leo A, Biganzoli L (2013). Utility of Gonadotropin-Releasing Hormone Agonists for Fertility Preservation in Young Breast Cancer Patients: The Benefit Remains Uncertain. Ann Oncol.

[85] Chen H, Xiao L, Li J, Cui L, Huang W (2019). Adjuvant gonadotropin-releasing hormone analogues for the prevention of chemotherapy-induced premature ovarian failure in premenopausal women. Cochrane Database Syst Rev.

